# Images of Extremely Rare Cantrell Phenomenon

**DOI:** 10.3390/diagnostics14101003

**Published:** 2024-05-13

**Authors:** Artur Fabijan, Sara Korabiewska-Pluta, Tomasz Puzio, Bartosz Polis, Tomasz Moszura

**Affiliations:** 1 Department of Neurosurgery, Polish-Mother’s Memorial Hospital Research Institute, 93-338 Lodz, Poland; jezza@post.pl; 2 Department of Cardiology, Polish-Mother’s Memorial Hospital Research Institute, 93-338 Lodz, Poland; sara.korabiewska@gmail.com (S.K.-P.); tmoszura@wp.pl (T.M.); 3 Department of Diagnostic Imaging, Polish-Mother’s Memorial Hospital Research Institute, 93-338 Lodz, Poland; tomekpuzio@gmail.com

**Keywords:** Cantrell syndrome, ectopia cordis, tetralogy of Fallot, thoracoabdominal syndrome

## Abstract

We present a case of a neonate born with prenatal diagnosis of Cantrell syndrome and ectopia cordis. This extremely rare congenital disorder underscores the significant need for multimodality imaging to plan further management. The aim of the study was to present the thoracoabdominal syndrome using a three-dimensional computed tomography angiography. The CT scans confirmed complex intracardiac defects consisting of tetralogy of Fallot, total anomalous pulmonary venous return and persistent left superior vena cava. In conclusion, Cantrell syndrome necessitates a multidisciplinary approach, from the onset of the prenatal diagnosis followed by prompt medical imaging and surgical interventions after birth. The thoracoabdominal wall defect including complete ectopia cordis is an extremely rare disorder with a fatal outcome.

A female neonate was born at 37 weeks of gestation with a prenatal diagnosis of Cantrell syndrome (POC) and ectopia cordis. The cesarean section was performed for a 33-year-old woman due to rupture of the membranes and congenital disorder. The birth weight was 3100 g and the Apgar scores were seven at 1 min and nine at 5 min. The infant presented the complete displacement of the heart outside the thoracic cavity along with the omphalocele and supraumbilical abdominal wall covered with a transparent membrane. Early treatment included warm saline-soaked sterile gauze, followed by placing the neonate under the radiant warmer.

On the first day, the patient required mechanical ventilation and the administration of vasoconstrictor drugs to stabilize the cardiorespiratory system. The beating heart was fully visible outside the thoracic cavity, bent towards the left side of the chest wall. A transthoracic echocardiogram in standard and modified views was performed. However, even minimal pressure from the echocardiographic probe or any movement of the heart during the examination resulted in decreased cardiac output, rhythm disturbances, and oxygen desaturation of the blood. Consequently, the diagnosis of ectopia cordis was confirmed, along with intracardiac tetralogy of Fallot with concurrent total anomalous pulmonary venous return and persistent left superior vena cava. Given the diversity of diagnoses, a three-dimensional computed tomography angiography (CT 3D) was performed (Figure 1 and Figure 2). The cardiac imaging quantified the diameter and volume of the left and right ventricles and myocardial tissue density parameters as being within normal reference values, as well as verifying atrial and ventricular septal defects, pulmonary artery stenosis, overriding aorta and right-sided aortic arch with four branches, right common carotid artery, left common carotid artery, left subclavian artery and right subclavian artery (known as arteria lusoria). Furthermore, the chest computed tomography angiography (CCTA) revealed a persistent left superior vena cava and collector of the pulmonary venous confluence to the right atrium. The lungs and liver were partially visible outside the thoracic and abdominal cavity due to an omphalocele.

Due to the complexity of the pathology, limited surgical possibilities and poor prognosis, a multidisciplinary team disqualified the patient from further treatment and withheld life-sustaining therapies. She died 7 days after birth due to cardiorespiratory failure.

POC also known as thoracoabdominal syndrome is an exceedingly rare congenital disorder characterized by defects of the heart, lower sternum, anterior diaphragm, and abdominal wall [1]. The hypothesis underlying this condition is based on abnormal mesodermal development at a very early stage of embryonic life [2]. Chromosomal analysis in this specific case revealed a normal female karyotype. Ectopia cordis is rarely associated with chromosomal abnormalities [3]. The cause of the disorder remains unknown, though there is a noted weak association with trisomy 18, and several cases are linked to other triploidies and familial inheritance associated with the X chromosome. The incidence rate is between 5.5 and 7.9 per million live births [4].

The intracardiac anomalies described in the literature may include ventricular septal defect, atrial septal defect, pulmonary stenosis or atresia and ventricular diverticulum. The combination of ectopia cordis with complex heart defects in the patient significantly worsened the prognosis. In the first days of life, treatment involves surgical, palliative repair of the abdominal hernia and diaphragmatic defect, as well as palliative or corrective repair of cardiovascular abnormalities. However, mortality in complex operations performed in very severe cases remains high [5]. Multiple surgical interventions should be avoided due to the high risk of wound infection [6].

The management of the thoracoabdominal syndrome requires complex, multidisciplinary care, encompassing both the medical challenges and emotional support for the patients and their families. In the case of this rare disorder, each case is unique and necessitates an individualized approach, focusing on maximizing quality of life and developmental potential. Advances in medicine and surgery offer new treatment possibilities, yet psychosocial support remains crucial to assist in managing life with such a serious diagnosis. In the face of these challenges, the role of the medical and scientific community in further investigating POC, its causes, and potential therapies is invaluable, aiming not only to improve care but also to enhance awareness and understanding of this complex condition.

## Figures and Tables

**Figure 1:**
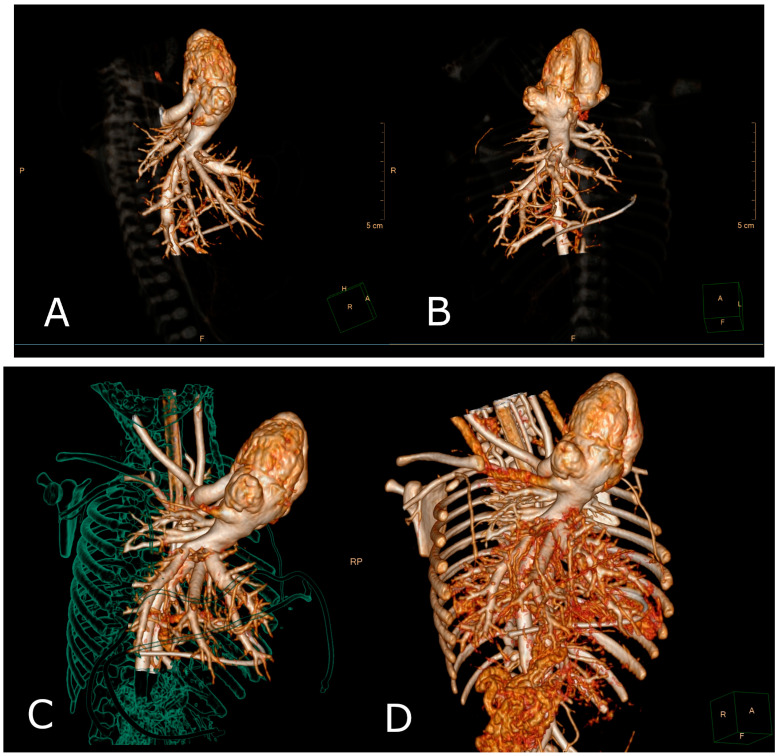
A three-dimensional computed tomography angiography (CT 3D) image revealed the entire heart external to the thoracic cavity with the apex oriented superiorly to the left-lateral view (**A**) and anterior view (**B**). (**C**) The 3D-volume rendering of the thoracic cavity with relation to vital organs and the lack of anterior coaptation of the chest wall components. (**D**) This image demonstrated mediastinal vascular malformations surrounded by thoracic vertebrae, short, hypoplastic ribs and sternal cleft.

**Figure 2:**
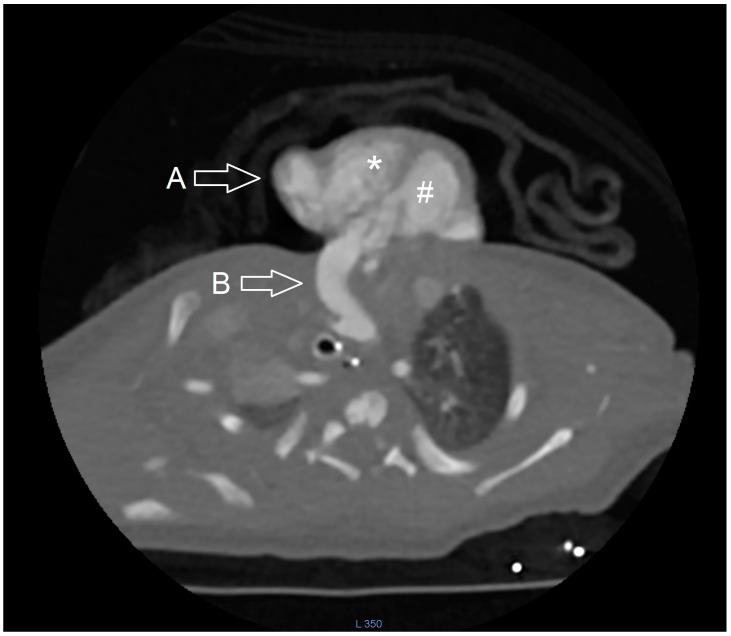
Axial chest computed tomography angiography (CCTA) scan showed a complete protrusion of the heart without the pericardium through the anterior chest wall. Arrow A—two-chamber long axis view of ectopia cordis. Arrow B—The aorta is positioned directly over the ventricular septal defect, passing into the right-sided aortic arch and then the descending aorta, which passes to the mediastinum. *—right ventricle, #—left ventricle.

## Data Availability

Not applicable.
